# P-473. Posaconazole therapeutic drug monitoring: the key to an effective antifungal prophylaxis

**DOI:** 10.1093/ofid/ofaf695.688

**Published:** 2026-01-11

**Authors:** Maria Alvarez de Toledo, Natalia A Mendoza-Palomar, Sonia Garcia Garcia, Maria Blanca Guembe Zabaleta, Laura Barenblit, Maria Larrosa-García, Laura Roig Soria, Berta Renedo Miró, Pere Soler-Palacín, Aurora Fernandez Polo

**Affiliations:** Vall d'Hebron University Hospital, Barcelona, Catalonia, Spain; Vall d'Hebron University Hospital, Barcelona, Catalonia, Spain; Vall d'Hebron University Hospital, Barcelona, Catalonia, Spain; Vall d'Hebron University Hospital, Barcelona, Catalonia, Spain; Vall d'Hebron University Hospital, Barcelona, Catalonia, Spain; Vall d'Hebron University Hospital, Barcelona, Catalonia, Spain; Vall d'Hebron University Hospital, Barcelona, Catalonia, Spain; Vall d'Hebron University Hospital, Barcelona, Catalonia, Spain; Vall d'Hebron Barcelona Hospital Campus, Barcelona, Catalonia, Spain; Vall d'Hebron Univeristy Hospital, Barcelona, Catalonia, Spain

## Abstract

**Background:**

Posaconazole suspension (POSAsusp) is widely used for the prophylaxis of invasive fungal infections (IFI) in children, however, the optimal pediatric dosing regimen remains undefined. Furthermore, due to its low bioavailability and high variability, therapeutic drug monitoring (TDM) is necessary. Our aim is to describe TDM of POSAsusp, identify factors that affect pharmacokinetics and evaluate its safety and effectiveness.

**Methods:**

Retrospective observational study including patients ≤18 years of age that received prophylactic POSAsusp during ≥1 week and with ≥1 TDM between 2017-2023. Posaconazole plasma trough concentrations (Ctrough) were targeted at 0.7-2.5 μg/ml.

**Results:**

Seventy-one patients were included, of whom 48 (67.6%) were male. The median age was 4.8 years (IQR 2.4-8.9 years). The most frequent underlying causes were primary immunodeficiencies (32.4%) and acute lymphoblastic leukemia (19.7%). Fifty-five (77.5 %) patients received an HSCT. A total of 373 Ctrough were obtained, and only 178 (47.8%) fell within the established therapeutic range. Of these, 187 (50.1%) were considered sub-therapeutic, while the remaining 8 (2.1%) were supra-therapeutic. Although there was no significant correlation with age group, the dose required in patients under 2 years of age was higher than with the rest of age groups (17.6 mg/kg/day vs. 12.8 mg/kg/day). Gastrointestinal disorders, concomitant use of PPIs and HSCT were significantly associated with subtherapeutic Ctrough. There were six patients (8.5%) with breakthrough IFI (2 A.fumigatus, 2 Candida spp., 1 Scopulariopsis spp., 1 without microbiological identification), and they were associated with subtherapeutic Ctrough (70.8% vs. 48.7%, p< 0,05). 2/6 patients died due to breakthrough IFI. Drug-related toxicity resulted in drug discontinuation in 4 patients (5.6%) (hypertransaminasemia, vomiting, erythroderma), 1/4 had supratherapeutic Ctrough.

**Conclusion:**

TDM is essential in patients receiving POSAsusp as antifungal prophylaxis as it ensures that patients obtain target Ctrough in order to prevent breakthrough IFI. It is especially necessary in patients with risk factors such as gastrointestinal disorders or PPI use. Further research is required to establish optimal dosing for patients under 2 years of age.

**Disclosures:**

Natalia A. Mendoza-Palomar, n/a, Gilead Sciences: Honoraria|Gilead Sciences: Inscription, travel and accomodation for infectious diseases meetings|Pfizer: Advisor/Consultant|Pfizer: Honoraria Pere Soler-Palacín, MD, PhD, MSc, Astellas: Research grants|CLS Behring: Grant/Research Support|Gilead: Grant/Research Support|Grifols: Grant/Research Support|Pfizer: Grant/Research Support|Takeda: Grant/Research Support

